# Unexpected stability of the iron(II) complex by an asymmetrical Schiff base from Fe(III): structure, magnetic and Mössbauer investigations

**DOI:** 10.1098/rsos.241334

**Published:** 2025-01-08

**Authors:** Dawit Tesfaye, Mamo Gebrezgiabher, Jonas Braun, Taju Sani, Sebastien Diliberto, Pascal Boulet, Nandakumar Kalarikkal, Christopher E. Anson, Annie K. Powell, Madhu Thomas

**Affiliations:** ^1^ Department of Industrial Chemistry, College of Natural and Applied Sciences, Addis Ababa Science and Technology University, PO Box 16417, Addis Ababa, Ethiopia; ^2^ Nanotechnology Center of Excellence, Addis Ababa Science and Technology University, PO Box 16417, Addis Ababa, Ethiopia; ^3^ Department of Chemistry, College of Natural Sciences, Salale University, PO Box 245, Fitche, Ethiopia; ^4^ Institute of Inorganic Chemistry (AOC), Institute of Nanotechnology (INT) and Institute for Quantum Materials and Technologies (IQMT), Karlsruhe Institute of Technology (KIT), Kaiserstr. 12, Karlsruhe 76131, Germany; ^5^ Institute Jean Lamour (IJL), UMR 7198, CNRS-Université de Lorraine, Nancy 54011, France; ^6^ School of Pure and Applied Physics, Mahatma Gandhi University, Kottayam, Kerala 686560, India

**Keywords:** low spin Fe(II) complex, asymmetrical Schiff base, Mössbauer spectroscopy

## Abstract

The asymmetric Schiff base prepared *in situ* from ethylenediamine and pyridine-2-carboxaldehyde reacts with Fe(ClO_4_)_3_·6H_2_O to form the Fe(II) complex [FeL_2_](ClO_4_)_2_ with L = *N*,*N*-diethyl-*N*′-(pyridin-2-yl)methylene)ethane-1,2-diamine, where the Fe(III) starting material has been unexpectedly reduced to Fe(II). This complex was characterized by elemental analysis, infrared spectra, single crystal and powder X-ray diffraction measurements, variable temperature DC magnetic measurement and room temperature Mössbauer spectroscopy. The asymmetric ligand L coordinates in a tridentate fashion through its pyridyl, azomethine and amino nitrogen atoms, generating a distorted octahedral geometry around the central metal ion. Variable temperature magnetic studies and a Mössbauer measurement show that the iron is locked in the low spin Fe(II) states.

## 1. Introduction

Schiff bases, the condensation products of primary amines and carbonyl compounds, are versatile platforms for the preparation of coordination compounds with desired properties [1–3]. Schiff bases with nitrogen donors and their metal complexes have been extensively investigated as a result of their structural diversity and electronic properties [4–6]. Among these, metal complexes with N6 octahedral coordination environment have attracted much interest in view of their structural as well as magnetic characteristics [7–9]. The great potential of Schiff base ligands can be used to achieve this goal, by selecting and connecting the required N donor bearing precursors (both aldehydes and amines) by an imine linkage and tuning their properties with appropriate ring substituents. In particular, iron(II) complexes with N6 coordination, are of interest in this context, as they can exhibit spin crossover (SCO) properties with appreciable hysteresis loops [10–13].

In our earlier reviews, we have shown, how the ligand field created by N6 coordination tune the SCO properties in iron(II) Schiff base complexes [9,14]. Also in our recent reports, we have shown Fe(II) complexes with N6 coordination from the Schiff bases obtained from pyridine-2,5-dicarboxaldehyde and various N coordinating aromatic primary amines, leading to the locking of the low spin (LS) state in the complexes [15,16] and also SCO Fe(III) compounds with N_2_O_4_ coordination having large hysteresis width [17].

The Schiff base from pyridine-2-carboxaldehyde and ethylenediamine and it is derivatives are of interest as it forms both symmetric [18] and asymmetric complexes [8,18–20]. In the asymmetric coordination case, either tridentate mono- or bis-chelation [8,18–20] occurs either with N3 coordination, from one ligand moiety along with co-ligands or N6 coordination from two ligands, respectively. In the symmetric ligation mode, both the azomethine and pyridyl nitrogens are coordinated from one ligand moiety along with two co-ligands [18].

In the ethylenediamine and pyridine-2-carboxaldehyde asymmetric Schiff base complexes in the tridentate N3 ligation mode, there are examples with a Cu(II) complex reported by Kang *et al.* [19]*,* and a Ni(II) complex by Banerjee *et al*. [18]. In Kang’s report, one ligand moiety is coordinated in a tridentate fashion through pyridyl and azomethine nitrogens and an amino group along with two bromo co-ligands, while in Ghosh’s report two ligand units wrap around the Ni(II) generating an octahedral geometry around the central metal ion. In the symmetrical case, one ligand moiety is coordinated to Ni^2+^ in a tetradentate fashion through both the pyridyl and azomethine nitrogens along with two aqua ligands resulting in an octahedral geometry [5,20].

Furthermore, spectroscopic data of the Fe(II) complex were reported by Baggio-Saitovitch & De Paoli and Jensen & Jorgensen [21,22], however, no crystallographic evidence was reported. It is noteworthy that in both cases, the synthesis of the complex was performed using Fe(II) starting materials. This is in contrast to the procedure presented here which starts with Fe(III) perchlorate under aerobic conditions and unexpectedly led to the previously reported Fe(II) complex. We report here the crystal structure and spin state investigation of this Fe(II) complex which was obtained by the *in situ* reduction of Fe(III) to Fe(II) and discuss the relative stability of iron oxidation states and the underlying reasons for this reduction.

Although Fe has a large range of oxidation states, in terms of accessible and common states, these are the Fe(III) and Fe(II) states. Octahedrally coordinated compounds of these states can both show SCO behaviour [23–26], i.e. for Fe(III) t_2g_^3^ e_g_^2^ to t_2g_^5^ e_g_^0^ and for Fe(II) t_2g_^4^ e_g_^2^ to t_2g_^6^ e_g_^0^. Although Fe(III) might be thought to be the more stable of these ions, the standard reduction potential for Fe(III)+e^−^ → Fe(II) shows that Fe(II) is the thermodynamically more stable because the reduction potential is +0.77 V. Indeed, this reduction was the original energy source for bacteria through a process called chemosynthesis. These iron-reducing bacteria preceded the development of photosynthesis as an energy source. The by-product of the overall photosynthesis reaction, O_2_, was the driving force for the Great Oxidation Event leading to the stabilization of higher oxidation state metal ions including large quantities of iron oxides and oxyhydroxides [27,28]. The highly insoluble nature of these materials is used as an explanation for this apparent stability of the Fe(III) state which can be explained using the Nernst equation [29]. It was thus unexpected to have an Fe(III) high spin starting material leading to the exclusively Fe(II) LS complex, which we report in this article. This is in contrast to the situation in a compound we recently reported where we also started from Fe(ClO_4_)_3_ and found that the high-spin state of Fe(III) was stable over the whole temperature range of the measurements. This compound has an N_2_O_4_ coordination environment [30].

**Scheme 1.**
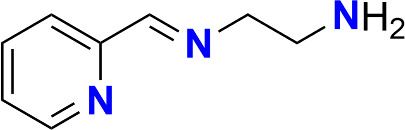
The ligand L = N.N-diethyl-N′-(pyridine-2-yl)methylene)ethane-1,2-diamine.

In order to steer the system towards Fe(III) SCO, we chose an exclusively nitrogen donating ligand. As mentioned above, this ligand (Scheme 1) was previously used to obtain similar complexes [8,18–20]. Here, however, we were successful in isolating an asymmetrical Schiff base complex of remarkably stable iron(II) by reacting Fe(ClO_4_)_3_∙6H_2_O with pyridine-2-carboxaldehyde and ethylenediamine under *in situ* conditions. This was subsequently characterized using single crystal X-ray diffraction (XRD) and various physico-chemical methods. Variable temperature magnetic and Mössbauer studies indicate that in the complex iron is in the +2 oxidation state and locked in the LS configuration over the whole measured temperature range. The spin state investigation is in line with the previously reported spectroscopic data [21,22] and confirms that we obtained the known Fe(II) complex from an Fe(III) starting material.

## 2. Material and methods

All chemicals and reagents were purchased from commercial sources and were of analytical reagent grade, used without further purification. *Caution! Although no such tendency was observed during the present work, perchlorate salts are potentially explosive and should be handled with care and in small quantities.* Fourier transform infrared (FTIR) spectra were measured on (Perkin Elmer Spectrum 400) in the 4000−400 cm^−1^ range. Elemental analyses were carried out on a CHNS-O analyzer (Flash 2000)—Thermo Scientific. The single-crystal XRD data were collected on a Bruker Kappa Apex II diffractometer equipped with a Mo-Kα IμS microfocus source (λ = 0.71073 Å). The Apex2 program package was used for the cell refinement and data reduction. The crystal was kept at 296(2) K during data collection. Using Olex2 [31], the structure was solved with the olex2.solve [32] structure solution program using Charge Flipping and refined with the SHELXL [33] refinement package using full-matrix least-squares refinement. All non-H atoms were assigned anisotropic thermal parameters; H-atoms bonded to C were placed in calculated positions. One of the perchlorate anions was disordered and refined using two sets of partial-occupancy oxygen atoms, applying similarity restraints to the Cl–O bond lengths and rigid-bond restraints to the thermal parameters.

Magnetic susceptibility measurements were carried out on an MPMS-3 SQUID magnetometer which can operate between 350 and 5 K, under an applied magnetic field of 0.1 T. Powder X-ray diffraction (PXRD) measurements were performed on Bruker D8 advance diffractometer in Bragg Brentano geometry and equipped with a Johansson Ge(111) monochromator and a Lynxeye position sensitive device (PSD) detector. The ^57^Fe Mössbauer spectrum was measured at 300K in transmission geometry with a ^57^Co/Rh source in constant acceleration mode; the velocity was calibrated against α-Fe. The evaluation of the Mössbauer spectra was performed by the least-square fitting of lines using the Winnormos (Wissel) program.

### 2.1. Synthesis of the complex [FeL_2_](ClO_4_)_2_ (1)

A solution of Fe(ClO_4_)_3_∙6H_2_O (0.354 g, 1 mmol) in 10 ml of methanol was added dropwise to a stirred methanolic solution (15 ml) of pyridine-2-carboxaldehyde (0.095 ml, 1 mmol) and ethylenediamine (0.676 ml, 1 mmol). The resulting mixture was stirred for a further 2 h. The brown solution was then filtered and allowed to evaporate slowly at room temperature (RT), during which the colour of the solution gradually turned from brown to blue. Blue block-shaped crystals suitable for XRD were isolated after two weeks. Yield: 0.68 g, 64%. Anal.: C_16_H_22_Cl_2_FeN_6_O_8_, Calcd.: C = 34.74, H = 4.00, *n* = 15.19, Found: C = 34.74, H = 3.98, *n* = 15.25%. FTIR (cm^−1^): 3325 (m), 3252 (m), 1602 (m), 1541 (m), 1461 (m), 1436 (m), 1077 (s), 903 (m), 878 (m), 781 (m), 620 (s), 530 (m), 496 (m), 455 (m) (electronic supplementary material, figure S2).

### 2.2. Single crystal structure analysis

**Figure 1.**
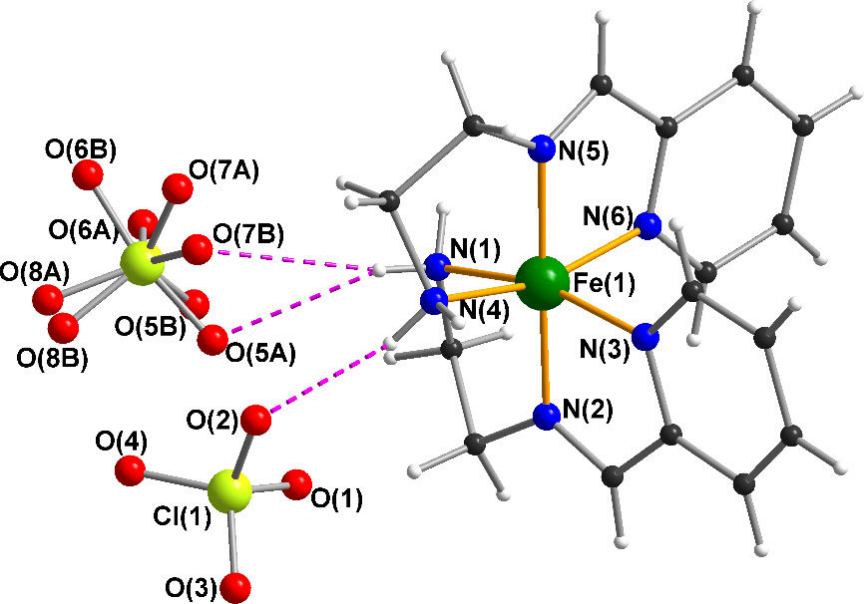
Labelled ball and stick drawing of the Fe(II) complex **1**. Colour code: Fe dark green; N blue; C grey; H light grey; Cl pale green and O red. Hydrogen bonds shown as purple dashed lines.

A single crystal suitable for structure determination was selected under an optical microscope, mounted on a goniometer head and centred on the Kappa Apex II diffractometer. The crystal was kept at 296 K during data collection. The structure was solved in the triclinic space group P$\overset{-}{1}$ (no. 2), with the following unit cell parameters: *a* = 8.2501(7) Å, *b* = 9.5781(9) Å, *c* = 14.5358(14) Å, *α* = 98.355(3)°, *β* = 91.642(3)°, *γ* = 102.642(3)°, *V* = 1106.70(18) Å^3^, *Z* = 2. Crystallographic data are summarized in table 1, and figure 1 displays the asymmetric unit. All data including observed and calculated structure factors have been deposited in the Cambridge structural database under the deposition number CCDC 2332654.

**Table 1.** Crystal data and structure refinement for complex.

empirical formula	C_16_H_22_Cl_2_FeN_6_O_8_
formula weight	553.14
temperature/K	296.15
crystal system	triclinic
space group	P−1
a/Å	8.2501(7)
b/Å	9.5781(9)
c/Å	14.5358(14)
α/°	98.355(3)
β/°	91.642(3)
γ/°	102.642(3)
volume/Å^3^	1106.70(18)
Z	2
ρ_calc_g/cm^3^	1.660
μ/mm^−1^	0.980
F(000)	568.0
crystal size/mm^3^	0.03 × 0.03 × 0.01
radiation	MoKα (λ = 0.71073)
2Θ range for data collection/°	4.412–56.564
index ranges	−10 ≤ h ≤ 10, −12 ≤ k ≤ 12, −19 ≤ l ≤ 19
reflections collected	28 219
independent reflections	5487 [*R*_int_ = 0.0503, *R*_sigma_ = 0.0372]
data/restraints/parameters	5487/90/346
goodness-of-fit on *F*^2^	1.025
final *R* indexes [I>=2σ (I)]	*R*_1_ = 0.0440, *wR*_2_ = 0.1043
final *R* indexes [all data]	*R*_1_ = 0.0634, *wR*_2_ = 0.1144
largest diff. peak/hole/e Å^−3^	0.58/–0.43

## 3. Results and discussion

### 3.1. Synthesis and characterization

Compound **1** was prepared by reacting hydrated ferric perchlorate with pyridine-2-carboxaldehyde and ethylenediamine under *in situ* conditions and crystallized by the slow evaporation of the resultant solution. Auto-reduction of the Fe(III) to Fe(II) occurs during the reaction in the presence of pyridine-2-carboxaldehyde; this is even more surprising when one considers that perchlorate is usually considered a strong oxidant. Liao *et al.* [34] have reported a similar case, where Fe(III) was reduced in the presence of various type of aldehydes. Attempts to synthesize the nitrate and chloro analogues with the same structural motif were unsuccessful. The asymmetrical ligand generated *in situ* is coordinated to the central Fe(II) in a tridentate fashion through the pyridyl, azomethine as well as the amino nitrogens. However, the nickel analogue having the same structural motif was reported by Banerjee *et al.* [18], and synthesized from the preformed ligand. This is in contrast to ours, which was obtained by *in situ* synthesis.

A suggested explanation for these observations on the present Fe system is that the high spin state of Fe(III) from the perchlorate salt starting material becomes LS on coordination by the ligand which is probably indicated by the initial brown colour of the reaction solution. The transition to a blue colour upon standing suggests the reduction towards Fe(II) which is then shown from the further experiments on the resulting blue crystals to be stable over a large temperature range *vide infra*.

From the Tanabe–Sugano diagrams, for high-spin Fe(III) to go to LS Fe(III) involves the ground state going from a ^6^S term to a ^2^I term, whereas for the Fe(II) it is a ^5^D state that goes to a ^1^I state [35]. In terms of ligand field considerations, we can suggest that the extra stabilization energy gained from the t_2g_^6^ e_g_^0^ configuration over the t_2g_^5^ e_g_^0^ is enough to stabilize the Fe(II) state. This is a result of the N6 coordination environment which prefers Fe(II) LS over Fe(III) high-spin. This in turn suggests to us that the initial formation of a LS Fe(III) state is the most plausible explanation.

### 3.2. Crystal structure

**Scheme 2.**
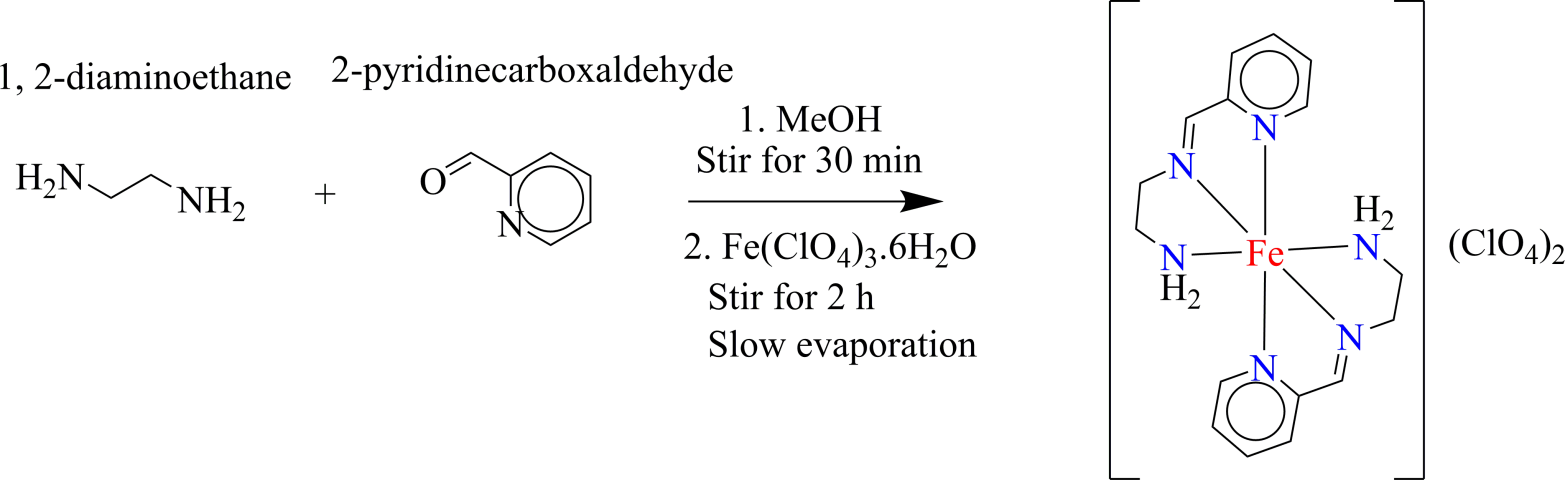
Synthetic route for the formation of [FeL_2_](ClO_4_)_2_

The Fe(II) complex was crystallized from the *in situ* reaction mixture by slow evaporation (Scheme 2). The compound crystallizes in the triclinic space group P$\overset{-}{1}$ with *Z* = 2. The asymmetric unit consists of one discrete [FeL_2_]^2+^ cation and two ClO_4_^−^ anions (figure 1, and Scheme 2). The entire unit cell is shown in the electronic supplementary material, figure S1.

In the hexa-coordinated Fe(II) complex, all the coordination sites are occupied by nitrogens with Fe–N bond lengths in the range 1.887(2)−2.024(3) Å, and the N–Fe–N angles are in the ranges 80.48(9)−98.36(9) and 163.42(9)−178.58(9)° (table 2). Consideration of the individual values shows that the shortest of these bonds involve the imine nitrogens, followed by those involving the pyridine nitrogens, with the two bond lengths to the amino groups being the longest. The two ligands chelate in a *mer*-fashion, but somewhat unusually the ligands are not in a mutually centrosymmetric arrangement; instead, the two amino nitrogens are *cis* to each other, as are the two pyridine nitrogens (table 2). These bond distances and bond angles are comparable with those in similar Schiff base complexes reported previously [19,36], and also consistent with Fe–N distances for LS–Fe(II) in the review by Zheng et al. [37].

**Table 2.** Crystal data and structure refinement for complex **1**.

N2—Fe1—N1	83.06 (10)	Fe1—N1	2.024 (3)
N2—Fe1—N3	80.89 (9)	Fe1—N2	1.887 (2)
N2—Fe1—N4	98.21 (9)	Fe1—N3	1.963 (2)
N2—Fe1—N5	178.58 (9)	Fe1—N4	2.016 (2)
N2—Fe1—N6	98.36 (9)	Fe1—N5	1.899 (2)
N3—Fe1—N1	163.95 (9)	Fe1—N6	1.974 (2)
N3—Fe1—N4	91.83 (9)		
N3—Fe1—N6	90.47 (8)		
N4—Fe1—N1	90.62 (11)		
N5—Fe1—N1	97.76 (10)		
N5—Fe1—N3	98.28 (9)		
N5—Fe1—N4	82.94 (10)		
N5—Fe1—N6	80.48 (9)		
N6—Fe1—N1	91.69 (10)		
N6—Fe1—N4	163.42 (9)		

Results of the SHAPE calculations [38] of complex **1** indicate the coordination geometry around the Fe(II) ion does not conform well to any idealized polyhedron, but can be best described as a strongly distorted octahedral coordination sphere (O_h_) with a minimum deviation from an ideal mode value of 7.601 (electronic supplementary material, table S1).

**Figure 2.**
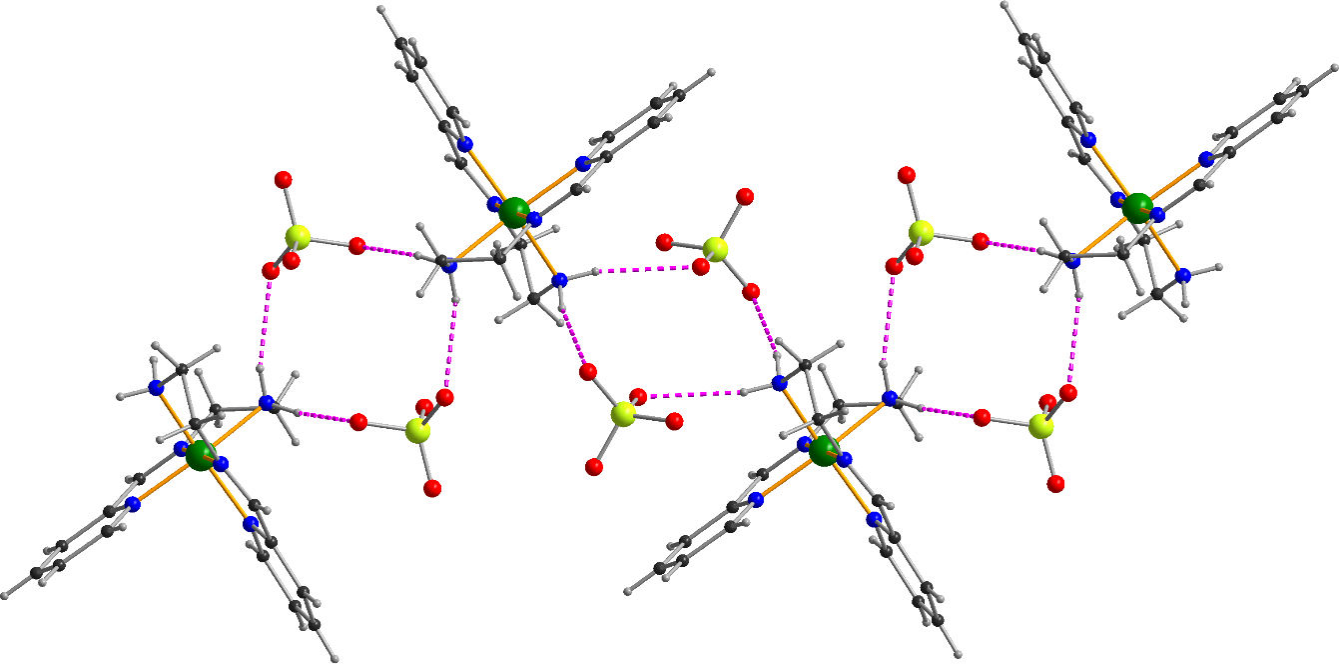
Zig-zag chain of Fe(II) complexes running parallel to the *c*-axis in the crystal structure of 1, showing the pairwise N–H∙∙∙O–Cl–O∙∙∙H–N linkages via the perchlorates. Only one of the disorder components of the second perchlorate is shown for clarity; hydrogen bonds are shown as purple dashed lines.

Within the crystal lattice, the complex molecules are linked into chains by hydrogen bonding between the amino N–H bonds and perchlorate anions (table 3). Pairs of N–H∙∙∙O–Cl–O∙∙∙H–N bridges link adjacent complexes within a chain (figure 2). Given the *cis*-arrangement of the two amino groups in a given molecule, these chains describe a zig-zag form. There are no significant intermolecular π–π interactions in **1**, however additional weak C–H∙∙∙O interactions further stabilize the structure.

**Table 3.** Selected hydrogen bond distances and angles in complex **1**. Symmetry operations: (i) 1 x, 1−y, 2−z; (ii) 1 x, 1−y, 1−z.)

atoms D,H,A	dist. D,H [Å]	dist. H,A [Å]	dist. D,A [Å]	angle D,H,A [°]
N1—H1A—O5A	0.84(3)	2.45(3)	3.151(12)	142.(3)
N1—H1A—O7B	0.84(3)	2.55(3)	3.378(11)	170.(3)
N1—H1B—O6Ai	0.87(3)	2.24(3)	3.060(9)	157.(3)
N1—H1B—O6Bi	0.87(3)	2.18(3)	3.014(8)	162.(3)
N4—H4A—O1	0.84(3)	2.27(3)	3.097(4)	166.(3)
N4—H4B—O2ii	0.88(3)	2.46(3)	3.311(4)	164.(3)

### 3.3. Powder X-ray diffraction analysis

The PXRD of the complex is in good agreement with the simulated one from the crystal structure as shown in the electronic supplementary material, figure S3. It proves the crystal structure is the representative of the bulk sample and excludes the possibility of multiple phases and thus confirms the purity of the sample.

### 3.4. Magnetic properties

The magnetic data are completely in line with a diamagnetic Fe(II) LS ground state over the whole temperature range up 350 K (electronic supplementary material, figure S4), which is consistent with the Mössbauer spectroscopy measurements at 300 K.

### 3.5. Mössbauer spectral studies

**Figure 3.**
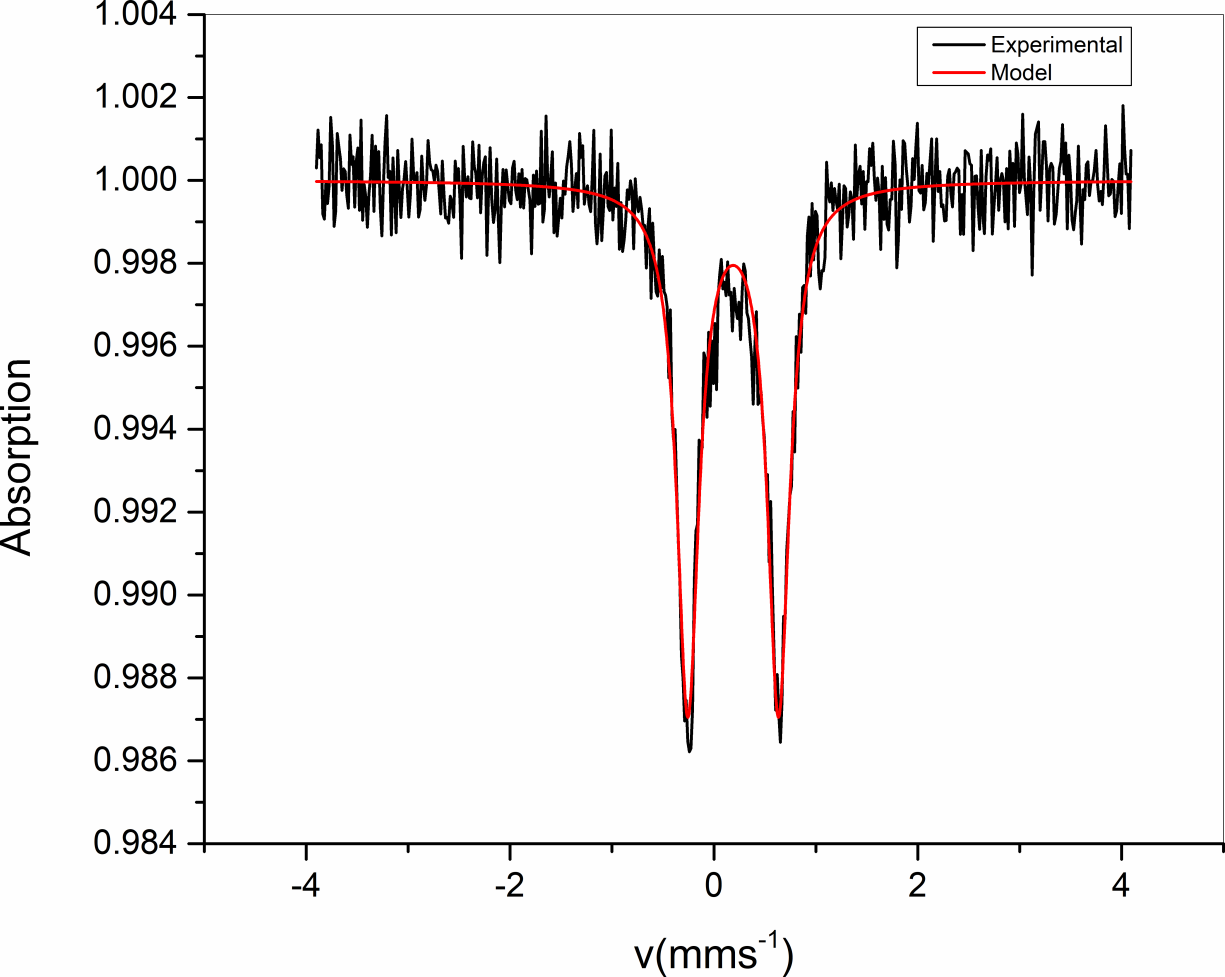
Room temperature ^57^Fe Mössbauer spectra of [FeL_2_](ClO_4_)_2_.

^57^Fe Mössbauer spectroscopy was carried out on the sample at RT and the result is presented in figure 3. The red line corresponds to the fit of the data.

The spectrum clearly highlights the presence of a doublet corresponding to a single iron site. The hyperfine parameters were determined with an isomer shift (IS) equal to 0.18 mm s^−1^ and a quadrupole splitting equal to 0.88 mm s^−1^. These parameters are consistent with an LS asymmetric Fe(II) complex reported by Brefuel *et al.* [39].

## 4. Conclusions

In summary, we have synthesized and characterized an asymmetrical Fe(II) Schiff base complex from pyridine−2-carboxaldehyde and ethylenediamine under aerobic *in situ* conditions. The complex was characterized by single crystal XRD, elemental analysis, infrared spectroscopy, SQUID magnetic measurement and Mössbauer spectroscopy. From the various physico-chemical studies, it has been established that the Schiff base formed during the reaction coordinates to the central metal ion in a tridentate *mer*-fashion through pyridyl, azomethine and amino nitrogens. It is interesting to note that reduction of Fe(III) to Fe(II) occurs during the reaction, forming LS Fe(II), even in the presence of perchlorate and atmospheric oxygen. Within the crystal lattice, the molecules are linked into zig-zag supramolecular chains by pairs of hydrogen bonding bridges involving the perchlorate counteranions. The observed Fe–N bond lengths and bond angles are in the range 1.887(2)−2.024(3) Å, and 80.48(9)°−98.36(9)° 163.42(9)°−178.58(9)°, respectively, consistent with the LS state of the complex at RT. This is further confirmed by the variable temperature magnetic susceptibility study between 350 and 5 K consistent with a LS state throughout the measured temperature range. From the Mössbauer studies, the isomer shift value of 0.18 mm s^−1^ and a quadrupole splitting of 0.88 mm s^−1^ are also consistent with the LS state.

## Data Availability

The crystallographic dataset supporting this article has been uploaded in the electronic supplementary material and has been submitted to the Cambridge Crystallographic Data Centre, reference CCDC number 2332654. These data can be obtained free of charge via [40]. Additionally, the following figures and table such as electronic supplementary material, figures S1–S4 and table S1 can be obtained in the supplementary information [41].
